# Response Surface Methodology Approach to Evaluate the Effect of Transition Metals and Oxygen on Photo-Degradation of Methionine in a Model Wine System Containing Riboflavin

**DOI:** 10.1021/acs.jafc.2c05275

**Published:** 2022-12-13

**Authors:** Daniela Fracassetti, Davide Ballabio, Melissa Mastro, Antonio Tirelli, David W. Jeffery

**Affiliations:** † Department of Food, Environmental and Nutritional Sciences (DeFENS), Università degli Studi di Milano, Via G. Celoria 2, 20133 Milan, Italy; ‡ Department of Earth and Environmental Sciences, University of Milano-Bicocca, Piazza della Scienza 1, 20126 Milan, Italy; § Department of Wine Science and Waite Research Institute, The University of Adelaide, PMB 1, Glen Osmond, South Australia 5064, Australia

**Keywords:** light, volatile sulfur compound, methanethiol, dimethyl disulfide, catechin, caffeic acid

## Abstract

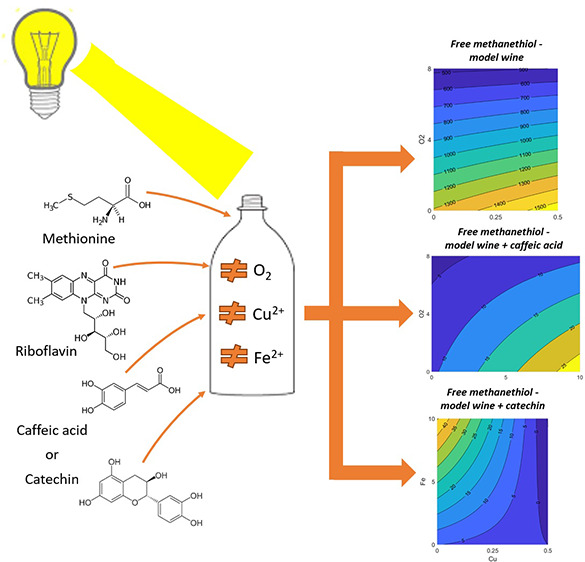

A Box–Behnken experimental design was implemented in model wine (MW) to clarify the impact of copper, iron, and oxygen in the photo-degradation of riboflavin (RF) and methionine (Met) by means of response surface methodology (RSM). Analogous experiments were undertaken in MW containing caffeic acid or catechin. The results evidenced the impact of copper, iron, and oxygen in the photo-induced reaction between RF and Met. In particular, considering a number of volatile sulfur compounds (VSCs) that act as markers of light-struck taste (LST), both transition metals can favor VSC formation, which was shown for the first time for iron. Oxygen in combination can also affect the concentration of VSCs, and a lower content of VSCs was revealed in the presence of phenols, especially caffeic acid. The perception of “cabbage” sensory character indicative of LST can be related to the transition metals as well as to the different phenols, with potentially strong prevention by phenolic acids.

## Introduction

**Figure 1:**
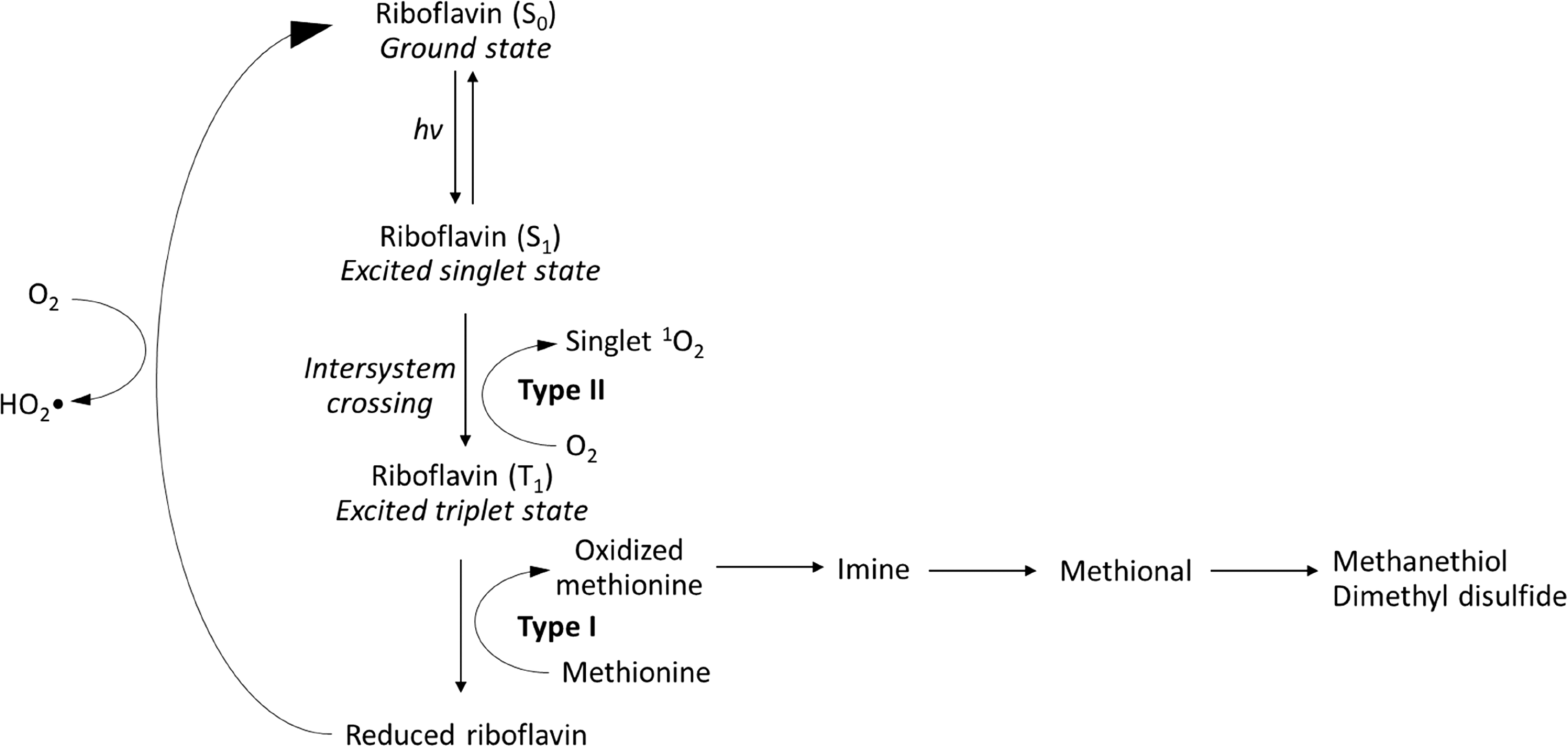
Reaction scheme showing photo-degradation of methionine in the presence of riboflavin to produce volatile sulfur compounds, among other products (adapted with permission from refs (25) (copyright 2019 Elsevier) and (35) (copyright 2020 American Chemical Society)).

Reactions due to light can have a detrimental effect on food quality, especially for products containing light-sensitive compounds known as photosensitizers. Vitamin B_2_ (riboflavin, RF) is a well-known example of such a constituent and is involved in photo-degradative reactions occurring in wine,^1^ milk,^2^ and beer.^3^ When exposed to light, especially at wavelengths from 370 to 450 nm, RF reaches an excited singlet state (S_1_) and, through an intersystem crossing, passes to a triplet state (T_1_). In this step, any oxygen present will be converted to its singlet form (simultaneously yielding ground state photosensitizer), with S_1_ oxygen being an electrophilic species responsible for nonradical reactions involving compounds such as alkenes, sulfides, and amines (Type II mechanism).^4,5^ Alternatively, excited T_1_ RF undergoes reduction, acquiring two electrons from compounds such as the amino acid methionine (Met) (Type I mechanism). The corresponding oxidation of Met yields methional, an unstable and light-sensitive molecule, which produces methanethiol (MeSH) and acrolein through a retro-Michael reaction (Figure 1).^6^

Upon further oxidation, two molecules of MeSH can yield dimethyl disulfide (DMDS),^7^ with these two types of volatile sulfur compounds (VSCs) having relatively low odor detection thresholds in wine (2–10 μg/L for MeSH and 20–45 μg/L for DMDS in white wine) and being responsible for odors reminiscent of cooked cabbage, onion, and garlic (reviewed in ref (8)). Furet et al.^9^ recently suggested that DMDS originates from a dimeric radical cation species, with the reaction occurring in a short time and without oxidizing species. Disulfides and polysulfides, whether symmetric or asymmetric, can be generated in the presence of copper(II) due to the reaction between hydrogen sulfide and thiols (e.g., MeSH).^10,11^

The appearance of unpleasant sulfur notes in wine can be associated with light-struck taste (LST),^6,12^ which is a concern when packaging wine in colorless (flint) glass bottles due to the extent of light transmission.^13^ The content of RF in wine is around 150–200 μg/L and mainly related to the strain of *Saccharomyces cerevisiae* used for alcoholic fermentation,^1,14^ whereas that of Met approaches 3–4 mg/L.^15−17^ Besides RF, wine contains other compounds sensitive to light, such as tartaric acid, whose photo-degradation produces glyoxylic acid in the presence of iron,^18^ with subsequent browning phenomena occurring due to the formation of xanthylium cation pigments.^19^ Iron present in wine also catalyzes redox reactions and, along with copper, is involved in the oxidation of phenols to quinones.^20^ Moreover, iron takes part in the formation of Strecker aldehydes generated from the reaction between quinones and α-amino acids.^21^ To date, however, the effect of iron and copper on LST formation had apparently not been investigated.

Iron and copper in wine can participate in oxidative phenomena at relatively low abundance (i.e., μg/L to mg/L), with average concentrations varying according to grape and wine production variables.^22^ Copper is commonly used for the removal of “reduced” aroma defects from wine due to the formation of copper complexes with sulfhydryl compounds.^22^ However, these complexes may behave as dissolved species and do not seem to be stable over time,^23^ meaning that copper is not able to definitively remove sulfur compounds associated with the “reduced” aroma defect. Moreover, copper addition to remove sulfhydryls is ineffective under anoxic conditions.^24^ Indeed, to a certain extent, the presence of oxygen can limit the appearance of defects due to sulfur compounds or LST.^25^ Nonetheless, the proper management of oxygen is fundamental, especially for white wine production, since oxidative phenomena involving phenolics and the loss of desired aroma compounds, such as the varietal thiols, both need to be prevented.^26^

Based on the impact that iron, copper, and oxygen can have on oxidation and sulfide formation, the present study aimed to test the hypothesis that the combined effect of iron, copper, and oxygen would influence LST formation in a wine-like solution containing RF and Met. This was investigated using a response surface methodology (RSM) approach, with the same experimental design being applied in the presence of catechin and caffeic acid as model phenols found in wine. Analysis of a number of oxidation products and VSCs was undertaken, along with an assessment of the sensory profiles of the samples. To the best of our knowledge, this is first time that the combined effect of oxygen, iron, and copper on the formation of LST has been investigated. The major understanding of the conditions favoring the formation of unpleasant VSCs as a consequence of the light-mediated reactions between RF and Met allows the formation of an overall picture of complex photo-degradative mechanisms.

## Materials and Methods

### Chemicals and Reagents

Methanol (99.9%), ethanol (96%), acetonitrile (99.9%), RF (98%), citric acid (99.5%), tartaric acid (99.5%), copper sulfate pentahydrate (98%), iron sulfate heptahydrate (98%), magnesium sulfate heptahydrate (98%), 2-mercaptoethanol (99%), *o*-phthalaldehyde (>99%) (OPA), l-Met (98%), methionine sulfoxide (Met sulfoxide) (98.5%), methionine sulfone (Met sulfone) (99%), *d*_6_-dimethyl sulfide (*d*_6_-DMS) (99%), isopropyl disulfide (96%), DMDS (98%), dimethyl trisulfide (DMTS) (98%), catechin (98%), caffeic acid (98%), sodium hydroxide (98%), and hydrochloric acid (37%) were purchased from Merck (Darmstadt, Germany). All the chemicals were of analytical reagent grade, at a minimum. HPLC grade water was obtained from a Milli-Q system (Millipore Filter Corp., Bedford, MA, USA).

### Experimental Design and RSM

Experiments utilizing a Box–Behnken experimental design and RSM approach were set.^27^ Model wine (MW) solution samples were formulated with different levels of copper (as Cu^2+^), iron (as Fe^2+^), and oxygen, as outlined in Table 1, leading to 15 runs. The trials were carried out in MW (5.0 g/L tartaric acid and 12% ethanol (v/v), adjusted to pH 3.2 with 10 M sodium hydroxide solution) containing 200 μg/L RF and 3 mg/L Met. Freshly prepared aqueous solutions of iron (as iron sulfate heptahydrate) and copper (as copper sulfate pentahydrate) were added, singularly or in combination, in accordance to the Box–Behnken design. As required, the samples were dosed with iron (5 or 10 mg/L) and copper (0.25 or 0.5 mg/L), and oxygen was partially (3 mg/L) or completely (below the limit of detection, 0.015 mg/L) removed by purging the solutions with nitrogen for 1 or 2 h, respectively, as separately determined using Oxydots and OxySense 101 analyzer (OxySense Inc., Las Vegas, NV, USA). The highest concentrations of both transition metals were chosen based on the work of Danilewicz.^28^ The samples that were protected from light during preparation and before and after controlled light exposure by covering with aluminum foil were placed in hermetically sealed clear glass bottles (100 mL), and stored in the dark at 20 ± 2 °C until required. The samples were exposed to fluorescent light for 2 h using light bulbs (Philips) emitting cold light (6500 K) with a luminous flux of 4000 lm, power of 65 W (26 × 8.8 cm), and high emission in the absorption wavelengths of RF (370 and 440 nm). The illumination involved a special triangle-shaped apparatus of 40 cm per side with three lamps placed on the top, at a distance of 20 cm.^25,29,30^ An analogous experimental plan was implemented with samples containing catechin (100 mg/L) or caffeic acid (70 mg/L), added from stock solutions prepared in methanol before the addition of the metals. Two samples were prepared for each treatment, with one being exposed to light and the other kept in the dark as a control.

**Table 1:** Concentration of Methionine Degraded, Acetaldehyde, Free Methanethiol (as *d*_6_-DMS Equivalents), Dimethyl Disulfide, Dimethyl Trisulfide, and Total VSCs, along with Sulfur Conversion Yield and Cabbage Sensory Score for Trials Performed in Model Wine Solution Containing Riboflavin (200 μg/L) and Methionine (3 mg/L) in the Presence of Absence of Copper, Iron, and Oxygen^a^

run	copper (mg/L)	iron (mg/L)	oxygen (mg/L)	degraded methionine (nmol/L)	acetaldehyde (mg/L)	methanethiol (nmol/L)	dimethyl disulfide (nmol/L)	dimethyl trisulfide(nmol/L)	total VSCs (nmol/L)	conversion yield (mol %)	sensory score
1	0	0	3	6308	nd	<2	114.50	62.17	416	6.6	6.6 ± 1.7^ac^
2	0.5	0	3	4557	nd	4.05	1432.69	139.95	3289	72.2	7.6 ± 0.9^a^
3	0	10	3	4180	nd	695.32	325.39	8.18	1371	32.8	7.0 ± 2.8^ac^
4	0.5	10	3	5461	nd	355.09	550.06	38.14	1570	28.7	6.6 ± 1.3^ac^
5	0	5	0	6717	nd	1116.57	592.74	44.14	2434	36.2	6.4 ± 1.5^abc^
6	0.5	5	0	8783	0.41	353.74	1127.75	0.00	2609	29.7	6.0 ± 1.2^abc^
7	0	5	8	6709	7.21	394.24	33.32	8.30	486	7.2	4.6 ± 1.9^bc^
8	0.5	5	8	3305	1.48	21.60	39.32	5.39	116	3.5	6.8 ± 1.3^ac^
9	0.25	0	0	8698	nd	18.90	1316.51	72.67	2870	33.0	6.2 ± 0.8^ac^
10	0.25	10	0	6276	0.41	40.50	1042.49	62.92	2314	36.9	7.8 ± 0.4^ab^
11	0.25	0	8	4905	nd	2.70	164.55	150.72	784	16.0	6.4 ± 2.1^ac^
12	0.25	10	8	2393	1.48	70.21	47.76	1.85	171	7.2	4.0 ± 2.2^bc^
13	0.25	5	3	6262	1.13	48.61	1012.02	93.49	2353	37.6	6.4 ± 0.5^abc^
14	0.25	5	3	7098	1.13	21.60	1220.37	80.97	2705	38.1	7.2 ± 1.1^abc^
15	0.25	5	3	6179	0.77	35.10	1357.00	108.29	3074	49.8	6.4 ± 0.5^abc^

a nd: not detected. Data are reported accounting for the difference between samples stored in the dark and those exposed to light. The sulfur conversion yield was estimated from the molar ratio of free sulfur compounds formed and methionine degraded. Different letters indicate significant differences between the sensory score means (*n* = 6) of the treatments (F test, α = 0.05).

### Determination of RF

The content of RF was assessed as described by Fracassetti et al.^1^ Briefly, the samples were filtered through a 0.22 μm PVDF filter (Millipore, Billerica, MA, USA) and analyzed with an Acquity UPLC (Waters, Milford, MA, USA) system equipped with a photodiode array (PDA) detector recording at 440 nm. Using an injection volume of 10 μL, separation was carried out with a Hypersil ODS C18 column (100 mm × 3.0 mm, 3 μm particle size, CPS Analitica, Milan, Italy) maintained at 25 °C with mobile phase A consisting of 90% (v/v) 50 mmol citrate buffer at pH 2.5 and 10% methanol, and mobile phase B containing 10% (v/v) 50 mmol citrate buffer at pH 2.5 and 90% methanol. The linear gradient increased from 0 to 70% B in 8 min at a flow rate of 0.6 mL/min followed by column washing with 100% B for 3 min and column conditioning with 0% B for 3 min. A six-point calibration curve was obtained for RF concentrations prepared in MW in the range 20–500 μg/L. Data acquisition and processing were performed with Empower 2 software (Waters).

### Determination of Methionine-Related Analytes

Met, Met sulfoxide, and Met sulfone concentrations were determined after derivatization with OPA as described by Fracassetti et al.^29^ with some modifications. The derivatization solution was prepared in a 10 mL volumetric flask by dissolving 250 mg of OPA in 1.5 mL of ethanol, adding 200 μL of 2-mercaptoethanol, and making up to volume with borate buffer (0.4 M at pH 10.5). The derivatization reaction was carried out at room temperature with 500 μL of borate buffer, 200 μL of sample, and 100 μL of OPA solution. The reaction mixture was vortexed for about 1 min, filtered with a 0.22 μm PVDF filter (Millipore), and injected after 5 min. Using an injection volume of 20 μL, analysis was performed with an Aquity UPLC coupled with a PDA detector recording at 338 nm. The column was a Waters Nova-Pak C18 (150 mm × 3.9 mm, 4 μm particle size). Mobile phase A was citrate buffer (10 mM, pH 7.5), and mobile phase B was acetonitrile/methanol/water (45/45/10 v/v/v). The linear gradient was 0–0.5 min, 5% B; 0.5–22 min, 47% B; and 22–24 min, 100% B, followed by column washing with 100% for 3 min and column equilibration with 5% B for 6 min. The flow rate was set to 1 mL/min, and the column temperature was 40 °C. Six-point calibration curves were obtained in the range 0.1–10 mg/L for each analyte in MW derivatized according to the method. Data acquisition and processing were performed with Empower 2 software (Waters).

### Determination of Acetaldehyde

Acetaldehyde concentration was determined with a colorimetric assay according to the OIV protocol^31^ with piperidine solution (10%, v/v) and sodium nitroferricyanide solution (0.4%, w/v) using a seven-point calibration curve (0–200 mg/L) of acetaldehyde in MW. The detection limit (LOD) of the method was 0.3 mg/L.

### Determination of VSCs

Free VSCs were analyzed by solid-phase microextraction (SPME)-GC/MS as reported by Fracassetti et al.^25,29,30^ Briefly, 5 mL of sample, 1.25 g of magnesium sulfate heptahydrate, 5 μL of isopropyl disulfide solution (12.5 mg/L in MW), and 20 μL of *d*_6_-DMS solution (125 mg/L in MW) were added to an SPME vial that was immediately capped and stored in the dark until the moment of analysis. Isopropyl disulfide was used as an internal standard to quantify DMDS and DMTS, and *d*_6_-DMS was used for MeSH. SPME was carried out with a 1 cm carboxen-polydimethylsiloxane-divinylbenzene fiber (50/30 μm, Supelco, Bellefonte, PA, USA) using a HTA autosampler (Brescia, Italy) fitted to an Autosystem XL gas chromatograph coupled with a Turbomass mass spectrometer (Perkin Elmer, Milan, Italy). The separation was achieved with a Stabilwax-MS column (30 m × 0.250 mm × 0.25 μm, Restek, Bellefonte, PA, USA) using helium as carrier gas at 1 mL/min. Electron ionization mass spectra (70 eV) were recorded in scan mode at *m*/*z* 33–350. Duplicate injections were carried out for each sample. Results for free MeSH are expressed relative to the concentration of *d*_6_-DMS (μg/L), whereas six-point calibration curves were prepared for DMDS and DMTS (0.5–200 μg/L). The estimated total VSCs corresponds to the sum (in moles) of the sulfur compounds detected. The estimated ratio between the moles of sulfur compounds formed (sum of free MeSH as *d*_6_-DMS equivalents, DMDS and DMTS concentrations) and the moles of sulfur lost as degraded Met and formed Met sulfox was determined.^25^ The odor activity values (OAVs) were determined as the estimated ratio between the amount of the VSC found in the sample and the respective perception threshold: MeSH, 0.3 μg/L; DMDS, 20–45 μg/L; and DMTS, 0.1 μg/L.^8^

### Sensory Analysis

Sensory evaluation was conducted on each trial for single samples of light-exposed and control MW samples. The panel consisting of six expert judges (average age 33, two females, and four males)^32^ carried out the olfactory scoring for “cooked cabbage” descriptor using a nine-point scale, with nine being the highest intensity (extremely perceived).^29,30^ MW spiked with Met (3 mg/L) and two different levels of RF (200 or 400 μg/L) and exposed to light for 4 h was used to train the panelists so they were confident about the perception of cooked cabbage note.^29,30^ The judges were calibrated by olfactory assessment of metal-free and phenol-free MW spiked with Met (3 mg/L) and RF (200 μg/L) exposed to light for increasing lengths of time up to 2 h, with the latter being considered to have a score of 5. The samples were presented to the panelists in a randomized order under ambient temperature and light. Coded ISO glasses containing 25 mL of sample and covered with a glass Petri dish were presented to the panelists.

### Statistical Analysis

Box–Behnken experimental outputs were analyzed using MODDE 6.0 (Umetrics). For the RSM approach, the fit of the model was evaluated by the coefficient of determination (*R*^2^). Partial least squares (PLS) was used to determine the regression models and establish the effects produced by the considered factors (copper, iron, and oxygen variables).^33^ The experimental variability was taken into account by means of the three replicated experimental runs executed in the center of the experimental domain. These replicates were then used in the ANOVA tests to evaluate and compare experimental and model variances. The significance of coefficients of factors (A, B, and C) and their interactions (AB, AC, and BC), as well as the presence of bias, were evaluated by means of ANOVA; only significant models are shown. Relationships between factors and predicted responses were then evaluated through the analysis of regression coefficients. Contour plots for response surface analysis were prepared considering the pairs of factors (among copper, iron, and oxygen) with the highest effect and/or interactions. The third factor that was not represented in the plot was considered at the average concentration (namely 0.25, 5, and 4 mg/L for copper, iron, and oxygen, respectively).

One-way ANOVA with post hoc Fisher’s LSD (α = 0.05) was carried out to determine the significant differences related to sensory analysis, using SPSS Win 12.0 program (SPSS Inc., Chicago, IL, USA).

## Results and Discussion

Photo-degradation reactions of RF and Met were evaluated to investigate LST phenomena in the presence of iron, copper, and oxygen. A Box–Behnken experimental design and the application of RSM were used for treatments involving different concentrations of iron (0, 5, and 10 mg/L), copper (0, 0.25, and 0.5 mg/L), and oxygen (0, 3, and 8 mg/L). This design was chosen to evaluate the possible impact of the three selected parameters on their own as well as their interactions. Moreover, the use of an RSM approach allowed for regression modeling (via PLS). Such models can be used to predict the behavior of the selected parameters providing information on how they can be managed in an oenological perspective. MW was adopted to avoid interferences from a multitude of matrix components present in wine and more accurately follow the light-induced reactions of RF and Met. The impact of caffeic acid and catechin, considered as model phenols for white wine, was also considered in the light-induced reactions between RF and Met. Phenols can act as antioxidants due to their hydroxyl groups leading to low redox potential and, consequently, increased oxygen consumption.^34^

### Effect of Transition Metals and Oxygen in MW

**Figure 2:**
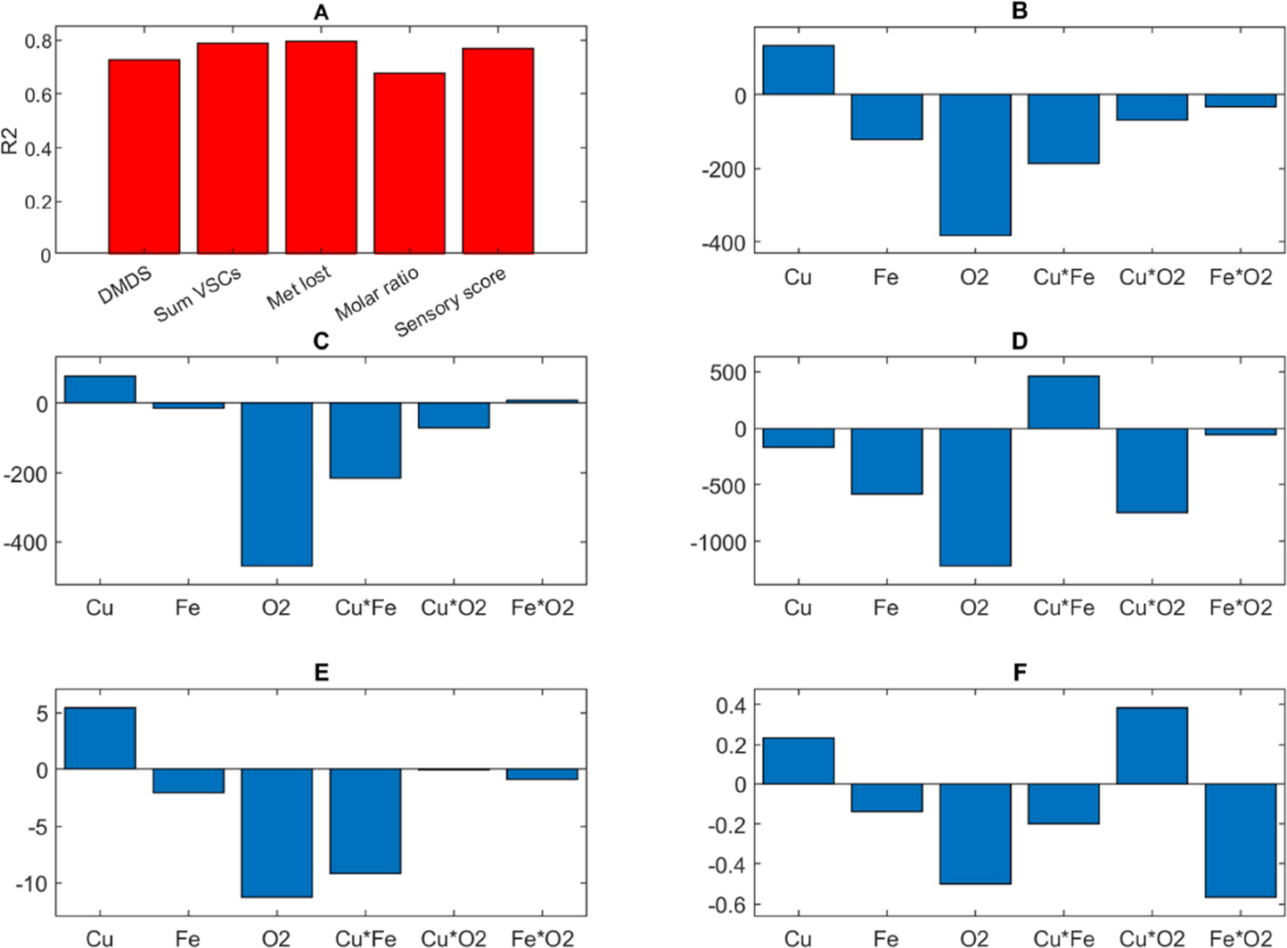
Results from photo-degradation trials carried out in model wine solution showing (A) PLS regression model performance (*R*^2^) and associated regression coefficients for (B) dimethyl disulfide (DMDS) concentration, (C) sum of VSCs, (D) methionine disappearance (Met lost), (E) molar ratio of sulfur compound formed/Met degraded, and (F) cabbage sensory score.

Exposing samples to light in these experiments caused the complete photo-degradation of RF such that it was not detected (data not shown), whereas samples maintained in the dark had an RF concentration of 203 ± 7 μg/L (initial concentration of 200 μg/L). The concentration of Met in the samples kept in the dark was 2.96 ± 0.59 mg/L (initial concentration of 3 mg/L), with greater decreases in all the light-exposed samples. The degradation of Met was predominant under anoxic conditions and in the presence of transition metals, corresponding to an average decrease of about 30% (Table 1). This was the same order of magnitude as that found previously for anoxic condition in the absence of transition metals.^25^ Met sulfone was not detected in any of the samples irrespective of light exposure, in accord with a previous study.^29^ Met sulfoxide, which can be produced from light-induced reactions in the presence of RF and Met,^29,35^ was detected in minor concentrations (0.10–0.15 mg/L) in runs 3, 4, 6, 10, 13, 14, and 15 (Table S1 of the Supporting Information). Evidently, Met sulfoxide formation may be limited by a greater presence of oxygen (i.e., not detected in samples containing oxygen equal to 8 mg/L) and favored in particular by iron at lower oxygen concentration, suggesting the major impact of the metal-catalyzed oxidation in comparison to oxygen-mediated oxidation. In any case, Met sulfoxide cannot explain the entire decrease in Met^29^ as other unknown compounds can be formed as a consequence of Met oxidation.^36^ The disappearance of Met could be explained by building PLS regression models calibrated on each modeled response, including DMDS concentration, sum of VSC concentrations, estimated molar ratio of sulfur compound formed/Met degraded, and cabbage sensory score. Models for these parameters were found to be significant on the basis of ANOVA and were associated with the following *R*^2^: DMDS concentration, 72.6%; total VSCs formed, 79.0%; Met lost, 79.6%; molar ratio of sulfur formed/Met degraded, 67.6%; and cabbage sensory score, 77.0%. Figure 2 shows bar graphs of the coefficients of determination (*R*^2^, Figure 2A) and the regression coefficients for each modeled response that will be discussed in turn, showing the disappearance of Met was negatively influenced by the presence of oxygen and the interaction of copper and oxygen, in particular (Figure 2D). Grant-Preece et al.^17^ and Fracassetti et al.^25^ reported a major decrease of Met under anoxic condition as Type I photo-degradation can only occur with Met acting as an electron donor. Moreover, the combination of copper and oxic condition could lead to the oxidation of other compounds (e.g., tartaric acid) that are more easily oxidizable than Met, and consequently, lower consumption of Met can occur. However, lower degradation of Met does not necessarily mean that lower concentrations of VSCs are formed, as presented later.

Acetaldehyde was determined because it can originate from ethanol oxidation by hydroxyl radicals induced by the Fenton reaction^37^ and was reported by Clark et al.^18^ in MW in the presence of tartaric acid and iron. The highest concentration of acetaldehyde was detected for the run where oxic condition was applied in the presence of iron (run 7, 7.21 mg/L; Table 1). The current data suggested that the decrease of Met did not lead to a major formation of acetaldehyde, with the latter mainly being affected by the presence of iron, copper, and oxygen. In fact, the decrease of Met was 1.00 mg/L in run 7 where acetaldehyde was present in the highest concentration, whereas the greatest decrease of Met was found in run 9 (1.30 mg/L) where acetaldehyde was not detected. Nonetheless, it was photo-induced reactions that triggered the oxidative mechanisms as no acetaldehyde was identified in the samples kept in the dark (Tables 1 and S1).

**Figure 3:**
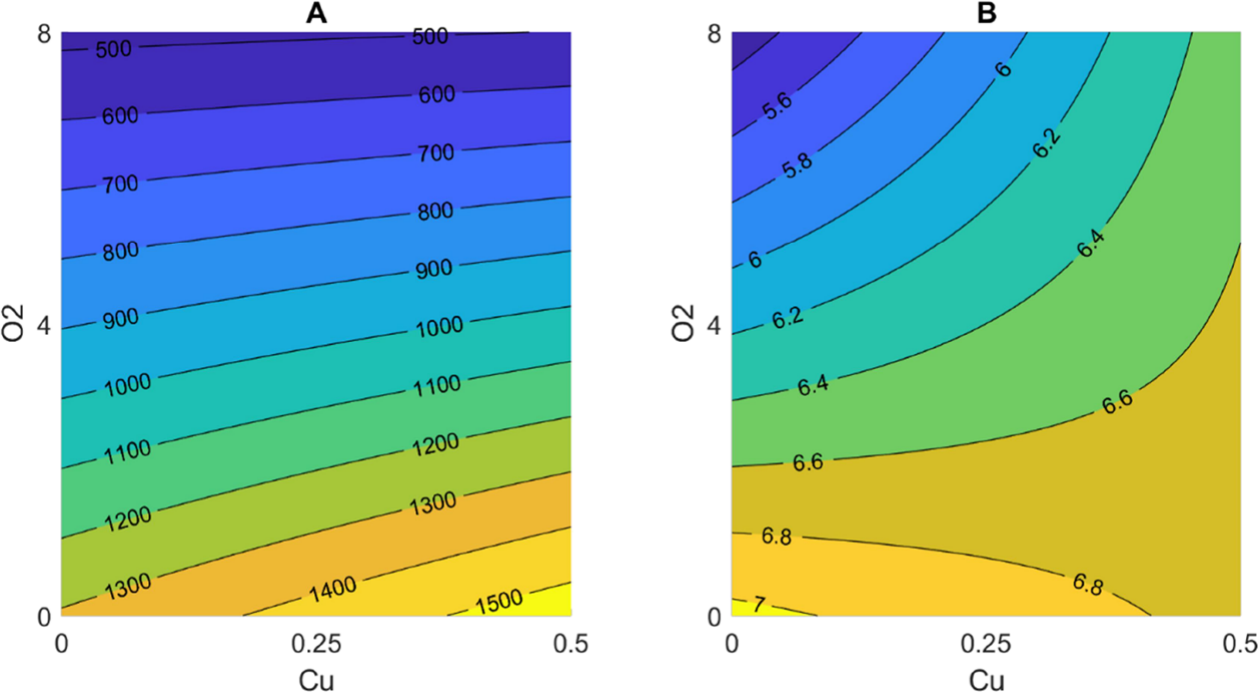
Results from photo-degradation trials carried out in model wine solution showing the contour plots for the interaction between oxygen and copper for (A) sum of VSCs and (B) cabbage sensory score.

In terms of VSC formation, the higher concentrations of one or more of free MeSH, DMDS, and DMTS were observed when the transition metals were present, singularly or in combination, with an oxygen concentration of 3 mg/L or under anoxic condition (runs 2, 5, 6, 9, 14, and 15; Table 1). Based on OAV, the highest OAVs for free MeSH were determined in the runs where iron was present alone (runs 3 and 5 with OAVs of 112 and 179, respectively; Table S2 of the Supporting Information) and it does seem to be partly affected by the concentration of dissolved oxygen (e.g., runs 6 and 7, Tables 1 and S1). The presence of both copper and iron affected the formation of free MeSH, and between the two transition metals, iron appeared to have a major impact (Table 1). Nonetheless, the regression model was not significant for free MeSH. An influence on DMDS formation may be evident considering that oxic conditions yielded among the lowest concentrations of this VSC (runs 7, 8, 11, and 12; Table 1). In particular, higher OAVs were determined in runs 2 and 11 (Tables S3 and S4) where copper was added, and relatively high values were also found when Cu and Fe were present (e.g., runs 13–15). This result indicated that copper does not allow the removal of VSCs and favored the development of DMDS and DMTS under the experimental conditions adopted, in accord with previous work.^38^ The formation of DMDS has been recently suggested to involve a dimer radical cation species^9^ that might not be bound by copper. DMTS could originate upon storage from the oxidation of methional and MeSH^39^ as well as due to the formation of H_2_S.^40^ With regard to DMDS, the total VSCs, and the molar ratio of sulfur formed/Met degraded, these were statistically significantly correlated only with oxygen (Figure 2B,C,E). As we found for Met decrease (Figure 2D), the regression coefficients were negative, as expected given that higher levels of VSCs can be detected in anoxic conditions.^25^ The contour plot evidenced the strong impact played by oxygen toward the formation of VSCs, which was even favored when copper was present at the highest concentration considered in this study (0.5 mg/L) (Figure 3A).

Finally, concerning cabbage sensory score, the variables with regression coefficients >0.5 were oxygen, copper and oxygen, and iron and oxygen (Figure 2F). The lowest cabbage sensory scores were found in runs 7 and 12 that were both characterized by oxic conditions (Table 1). The concentration of oxygen yielded a negative regression coefficient; the modeled perception of LST defect was lowered with higher oxygen concentration. Oxygen has the ability to act as a quencher of excited RF^41^ and can produce Met sulfoxide,^35^ a stable compound that cannot be oxidized further, thereby limiting the formation of sulfur compounds.^36^ The interaction of copper and oxygen was positively related to the sensory perception of LST. Indeed, the impact of copper appeared evident based on when the concentration of this metal was the highest (0.5 mg/L), whereby an increase of LST perception was observed even for the oxic condition (oxygen at 8 mg/L) (Figure 3B). Moreover, considering that only free MeSH was determined according to the method used, the presence of copper–MeSH complexes cannot be excluded, and although their direct effect on the perception of LST is unlikely, they do not seem to be stable over time.^23^ Copper is not able to complex with sulfur-containing compounds that lack a free sulfhydryl group such as disulfides,^42^ which themselves can form in the presence of copper.^23^ In fact, copper can form disulfides and polysulfides^11^ that are oxidizable by the presence of oxygen leading to a consequent copper-based oxidation of sulfur-containing compounds. Even if these compounds have higher perception thresholds in comparison to free MeSH, they cannot be removed and, consequently, LST persists.^29,43^ The interaction of iron and oxygen had a lowering effect on the modeled perception of LST.

### Effect of Transition Metals and Oxygen in the Presence of Caffeic Acid

As found in the earlier trials, light exposure of solutions containing caffeic acid also led to the complete degradation of RF (data not shown) compared to 225 ± 12 μg/L RF determined in the samples stored in the dark. In most of the cases, the degradation of Met may have been favored with light under anoxia (Table 2) and in the samples with oxygen at 3 mg/L (dark stored samples had 3.06 ± 0.44 mg/L Met). However, the extent of Met decrease was lower in magnitude in comparison to the runs in MW without phenolics, and up to about −4% on average, with the exception of run 6 (−0.61 mg/L), run 1 (−0.31 mg/L), and run 5 (−0.24 mg/L) (Table S5 of the Supporting Information). This result indicated the possible protective effect of caffeic acid toward Met degradation, possibly because caffeic acid can compete with Met in donating electrons to RF in a Type I mechanism as well as reacting with singlet oxygen in a Type II mechanism, as observed for gallic acid.^35^ Met sulfoxide was detected at up to 0.5 mg/L in the runs in oxic condition and where oxygen was 3 mg/L (runs 1, 3, 4, 5, 6, 13, 14, and 15; Table S5 of the Supporting Information), suggesting the major influence of oxygen on its formation even if a regression model could not be obtained with regard to Met sulfoxide formation. Trace amounts (<0.06 mg/L) of Met sulfone were detected only under oxic condition (data not shown), although this could be considered a negligible amount, being 10 times lower than Met sulfoxide.

**Table 2:** Concentration of Methionine Degraded, Acetaldehyde, Free Methanethiol (as *d*_6_-DMS Equivalents), Dimethyl Disulfide, Dimethyl Trisulfide, and Total VSCs, along with Sulfur Conversion Yield and Cabbage Sensory Score for Trials Performed in Model Wine Solution Containing Caffeic Acid (70 mg/L), Riboflavin (200 μg/L), and Methionine (3 mg/L) in the Presence of Absence of Copper, Iron, and Oxygen^a^

run	copper (mg/L)	iron (mg/L)	oxygen (mg/L)	degraded methionine (nmol/L)	acetaldehyde (mg/L)	methanethiol (nmol/L)	dimethyl disulfide (nmol/L)	dimethyl trisulfide (nmol/L)	total VSCs (nmol/L)	conversion yield (mol %)	sensory score
1	0	0	3	2072	0.36	<2	nd	nd	1.35	0.1	4.3 ± 1.5^ab^
2	0.5	0	3	127	0.36	<0.1	10.41	20.81	84.60	66.7	6.3 ± 1.7^abc^
3	0	10	3	124	1.07	25.65	<1	nd	27.09	21.9	5.3 ± 2.5^abc^
4	0.5	10	3	833	–1.79	9.45	3.60	<0.8	17.09	2.1	4.8 ± 1.5^abc^
5	0	5	0	1579	2.15	43.20	1.20	nd	45.61	2.9	4.8 ± 2.1^abc^
6	0.5	5	0	4084	–1.43	9.45	1.68	<0.8	14.63	0.4	5.5 ± 1.3^abc^
7	0	5	8	128	7.51	4.05	<1	nd	4.37	3.4	4.0 ± 1.6^b^
8	0.5	5	8	772	0.72	2.70	<1	nd	3.02	0.4	6.5 ± 1.0^ac^
9	0.25	0	0	1203	0.72	<2	1.60	10.19	35.12	2.9	7.0 ± 0.8^c^
10	0.25	10	0	913	0.36	28.35	<1	<0.8	30.06	3.3	6.3 ± 1.5^abc^
11	0.25	0	8	711	1.07	4.05	<1	1.43	8.65	1.2	5.8 ± 1.0^abc^
12	0.25	10	8	759	2.15	6.75	nd	nd	6.75	0.9	5.0 ± 1.4^abc^
13	0.25	5	3	669	2.15	13.50	<1	<0.8	14.30	2.1	4.7 ± 1.2^abc^
14	0.25	5	3	872	2.50	16.20	<1	<0.8	17.00	2.0	6.3 ± 1.0^abc^
15	0.25	5	3	740	2.00	18.90	<1	<0.8	21.41	2.9	5.3 ± 2.2^abc^

a nd: not detected. Data are reported accounting for the difference between samples stored in the dark and those exposed to light. The sulfur conversion yield was estimated from the molar ratio of free sulfur compounds formed and methionine degraded. Different letters indicate significant differences between the sensory score means (*n* = 6) of the treatments (F Test, α = 0.05).

Acetaldehyde was detected in all the runs but primarily favored with light exposure, as observed by an increase under most of the conditions tested. Higher concentrations were determined in the runs where iron was present (Tables 2 and S5). Moreover, the highest formation of acetaldehyde was detected in run 7 (7.21 mg/L) under oxic condition and in the presence of 5 mg/L iron. As previously mentioned, the increase of acetaldehyde can be related to the Fenton reaction.^18^

**Figure 4:**
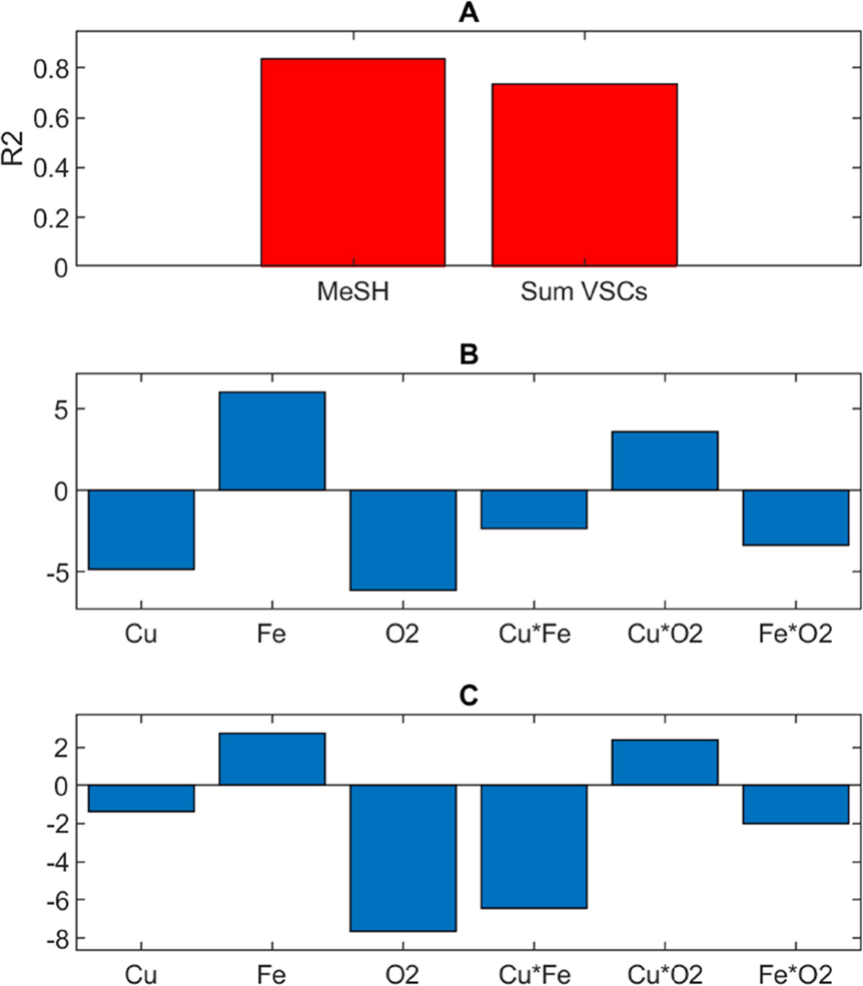
Results from photo-degradation trials carried out in model wine solution added with caffeic acid showing (A) PLS regression model performance (*R*^2^) and associated regression coefficients for (B) free methanethiol (MeSH) concentration and (C) sum of VSCs.

**Figure 5:**
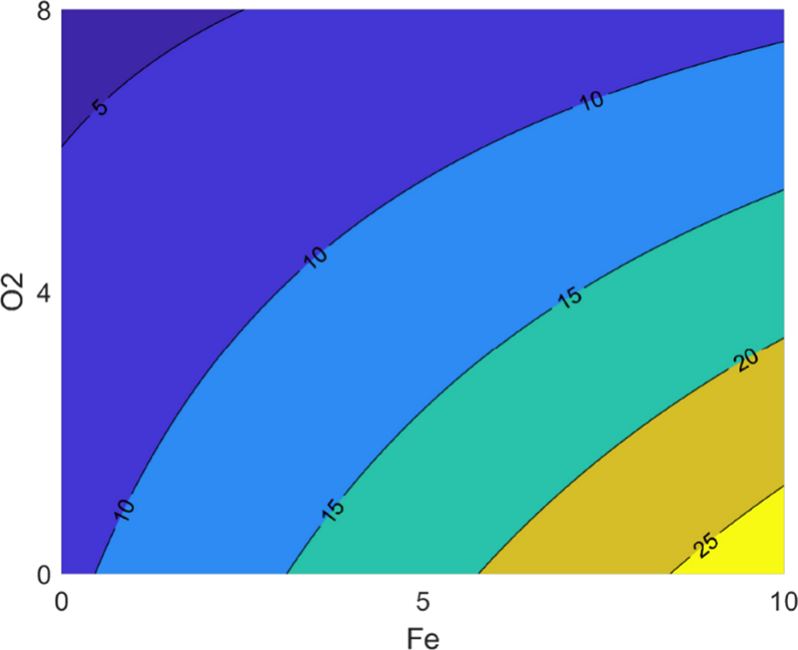
Results from photo-degradation trials carried out in model wine solution added with caffeic acid showing the contour plots for the interaction between oxygen and iron for free methanethiol concentration.

Concerning the formation of VSCs, in general, the most abundant VSC in experiments containing caffeic acid was free MeSH, with the exception of run 2, where DMTS was the highest (Table 2). Nonetheless, the presence of caffeic acid was found to markedly limit the formation of VSCs in comparison to MW (Table 1), further supporting the competition of this hydroxycinnamic acid against Met in donating electrons^35^ and potentially counteracting the release of VSCs. Moreover, the oxidized form of caffeic acid could also bind MeSH, limiting its oxidation to DMDS and the formation of DMTS. With regard to the polysulfides, it seems that copper alone could favor the formation of DMTS, given the highest concentrations were found in runs 2 and 9 (2.63 and 1.29 μg/L, respectively). As found in MW, the temporary effect of copper binding of VSCs^42^ may have also been evidenced, since free MeSH was detected even in the runs where copper was present at the highest level considered in this study (0.5 mg/L). Nonetheless, comparing runs 5 and 6, the relative concentration of free MeSH was lower in the latter (−78%), which differed by the addition of copper (0.5 mg/L). Iron played an important role in the formation of this VSC even at 5 mg/L (the average level investigated) (Table 2). The regression models yielded *R*^2^ values of 83.7% for free MeSH concentration and 73.7% for sum of VSCs and showed significant differences due to copper, iron, and oxygen for free MeSH (Figure 4A). Negative influence was found between free MeSH concentration and copper (Figure 4B). This finding was consistent with the ability to form Cu–S complexes by means of charge transfer between the two species; in this way, copper can be reduced and a proportion of the sulfur compound oxidized.^23^ The impact of iron with free MeSH concentration was positive (Figure 4B). As previously mentioned, this can be potentially due to Strecker degradation from which aldehydes originate, starting from α-amino acids such as Met. Iron can catalyze oxidative phenomena favoring the formation of methional, and consequently, of free MeSH. Oliveira et al.^44^ monitored the evolution of Strecker aldehydes and observed that the addition of copper and iron to MW under oxic condition generated reactive oxygen species (ROS), producing quinones. These unstable quinones, specifically those deriving from the oxidation of caffeic acid, can react with the α-amino acids to generate Strecker aldehydes, with the corresponding reduction of quinones back to phenols. Oxygen was modeled as a negative contributor to free MeSH concentration (Figure 4B) in agreement with Fracassetti et al.,^25^ where higher concentrations of VSCs were detected in MW under anoxic conditions. The interaction between oxygen and iron appears evident looking at their contour plot (Figure 5A): the decrease of oxygen and the increase of iron were responsible for higher concentrations of free MeSH. This suggests that the presence of iron in wine should be considered due to its impact on the occurrence of VSCs as reported herein as well as for the light-dependent formation of xanthylium ions.^19^ As shown in Figure 4C, a negative influence of oxygen was observed on the total content of VSCs determined. This provided further evidence of the strong impact of oxygen on VSCs due to the occurrence of Type I and/or Type II pathways,^17,25^ which was enhanced by the presence of caffeic acid (i.e., Table 2 vs Table 1). The impact of oxygen was borne out by the cabbage sensory scores being the lowest for run 7 (4.0 ± 1.6), which was characterized by oxic condition with iron at average level (5 mg/L), thus suggesting that caffeic acid can be an easier target for donating electrons. Such a hypothesis could be revealed by run 1 containing oxygen at 3 mg/L in the absence of transition metals, which also yielded a low cabbage sensory score (4.3 ± 1.5). In general, the cabbage sensory scores were lower in the presence of caffeic acid (Table 2) in comparison to those found for MW (Table 1). Copper and iron interaction was a negative contributor to the total content of VSCs, which suggested that the combined presence of the two transition metals can limit the formation of VSCs, even if higher levels of iron alone can contribute to an increase in concentration of free MeSH according to the model. This could be likely based on the ability of copper to oxidize MeSH to form disulfides and polysulfides.^10,11^ These sulfur-containing compounds have higher thresholds than that of MeSH. In terms of OAV in the presence of caffeic acid, values determined for free MeSH were up to 5 (Table S2 of the Supporting Information), those for DMDS were up to 1 (only detected in runs 2, 4, 5, 6, and 9) (Table S3 of the Supporting Information), and those for DMTS were up to 1 (Table S4 of the Supporting Information), with the exception of runs 2 and 9 in the latter case, where the OAVs were greater than 10. Even so, the values for DMTS across the runs were much lower than those found for MW (Table S4 of the Supporting Information), thus supporting the important impact of phenolics against the formation of VSCs. Nonetheless, the models were not significant in the presence of caffeic acid, suggesting its possible ability to prevent the appearance of LST.

### Effect of Transition Metals and Oxygen in the Presence of Catechin

Consistent with the trials in MW and in the presence of caffeic acid, no RF was found after light exposure in the system containing catechin (data not shown). In contrast, the RF concentration in the samples stored in the dark was 214 ± 9 μg/L. Anoxic condition and an average level of oxygen (3 mg/L) caused a major decrease of Met (compared to a concentration of 3.29 ± 0.29 mg/L for samples maintained in the dark). However, with an average Met degradation of about −15% (Table 3), the presence of catechin had less of a protective effect in comparison to caffeic acid (average Met decrease up to −4%; Table 2). Met sulfoxide was detected in the assay with catechin only in runs 10–15 (up to 0.3 mg/L; Table S6 of the Supporting Information), albeit lower in concentration compared to the experiments with caffeic acid. A negligible content of Met sulfone was found (<0.06 mg/L), again indicating the minor formation of Met sulfone in comparison to Met sulfoxide.

**Table 3:** Concentration of Methionine Degraded, Acetaldehyde, Free Methanethiol (as *d*_6_-DMS Equivalents), Dimethyl Disulfide, Dimethyl Trisulfide, and Total VSCs, along with Sulfur Conversion Yield and Cabbage Sensory Score for Trials Performed in Model Wine Solution Containing Catechin (100 mg/L), Riboflavin (200 μg/L), and Methionine (3 mg/L) in the Presence of Absence of Copper, Iron, and Oxygen^a^

run	copper (mg/L)	iron (mg/L)	oxygen (mg/L)	degraded methionine (nmol/L)	acetaldehyde (mg/L)	methanethiol (nmol/L)	dimethyl disulfide (nmol/L)	dimethyl trisulfide (nmol/L)	total VSCs (nmol/L)	conversion yield (mol %)	sensory score
1	0	0	3	3249	0.36	nd	nd	nd	nd		3.8 ± 1.9^ac^
2	0.5	0	3	2535	1.07	<2	32.11	44.32	198.55	7.8	6.8 ± 0.4^b^
3	0	10	3	2951	0.36	81.01	4.00	nd	89.02	3.0	7.6 ± 0.9^b^
4	0.5	10	3	3491	1.43	<2	4.40	nd	10.16	0.3	6.2 ± 1.1^ab^
5	0	5	0	2242	3.94	31.05	44.21	2.57	127.16	5.7	7.0 ± 1.2^b^
6	0.5	5	0	3089	–0.36	nd	nd	<0.8	0.21	0.0	5.2 ± 2.9^ac^
7	0	5	8	3193	3.94	37.80	1.20	nd	40.21	1.3	3.6 ± 1.5^c^
8	0.5	5	8	209	1.43	nd	2.64	<0.8	6.03	2.9	6.2 ± 1.9^a^
9	0.25	0	0	3041	0.00	<2	86.17	76.21	402.33	13.2	6.4 ± 2.1^b^
10	0.25	10	0	1290	0.72	8.10	14.98	1.32	42.01	3.3	6.2 ± 1.5^b^
11	0.25	0	8	54	–0.72	nd	4.97	6.16	28.42	52.8	5.2 ± 1.8^ac^
12	0.25	10	8	2467	2.50	4.05	11.61	<0.8	27.81	1.1	5.0 ± 1.4^ac^
13	0.25	5	3	608	1.07	<2	21.01	13.72	84.53	13.9	7.2 ± 1.1^b^
14	0.25	5	3	536	4.65	2.70	39.48	26.65	161.62	30.1	6.0 ± 1.2^b^
15	0.25	5	3	368	–1.07	nd	57.15	27.90	197.99	53.8	7.2 ± 0.4^b^

a nd: not detected. Data are reported accounting for the difference between samples stored in the dark and those exposed to light. The sulfur conversion yield was estimated from the molar ratio of free sulfur compounds formed and methionine degraded. Different letters indicate significant differences between the sensory score means (*n* = 6) of the treatments (F Test, α = 0.05).

Similar to the trials with caffeic acid, acetaldehyde was detected in all runs, including those kept in the dark. The highest concentration of acetaldehyde was detected in the runs where iron was present (in particular, run 7 with 3.94 mg/L; Tables 3 and S6 of the Supporting Information).

In most of the cases, the major role played by oxygen in terms of VSC concentrations was again highlighted, with their formation being limited under oxic condition in the presence of catechin (Table 3). The findings supported the notion that the presence of phenolics can counteract the formation of VSCs associated with LST; although among the two phenols, in most cases, the VSCs were higher in the presence of catechin (particularly for DMDS and DMTS). The two runs with the highest relative concentration of free MeSH were runs 3 and 7 (3.90 and 1.82 μg/L, respectively; Table 3). The latter also showed the highest cabbage sensory score for LST (Table 3). On the contrary, the lowest cabbage sensory score was found for run 7, in which DMDS was detected at low level (0.11 μg/L) and DMTS was not revealed. Nonetheless, such differences in VSC concentrations cannot explain the intensity perceived for LST. The phenols could suppress, accentuate, or show negligible effect on the perception of aroma compounds,^45^ and the presence of oxygen and transition metals could also modify the perception of LST. However, further studies are necessary to demonstrate these hypotheses. Free MeSH and DMTS were determined with the highest OAVs, being up to 10 in run 3 for MeSH and similar in runs 2, 9, 13, 14, and 15 for DMTS (Tables S2 and S4 of the Supporting Information). In contrast, DMDS showed OAVs between 0 and 1 in all runs (Table S3 of the Supporting Information). Therefore, free MeSH and DMTS were considered to be the VSCs having a greater impact on LST, even in the presence of catechin. The chemical nature of phenols could also play a role in the perception of LST; as in most of the cases, OAV values were higher with catechin in comparison to caffeic acid (Tables S2–S4 of the Supporting Information), but negligible differences in the cabbage sensory score were found.

**Figure 6:**
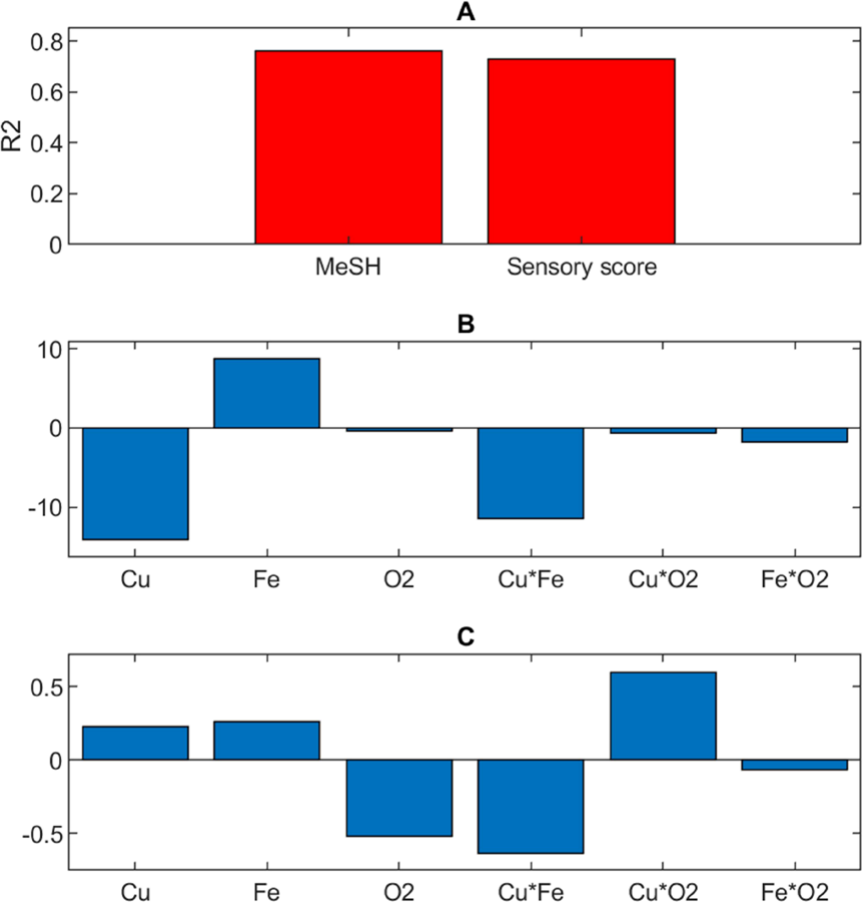
Results from photo-degradation trials carried out in model wine solution added with catechin showing (A) PLS regression model performance (*R*^2^) and associated regression coefficients for (B) free methanethiol (MeSH) concentration and (C) cabbage sensory score.

**Figure 7:**
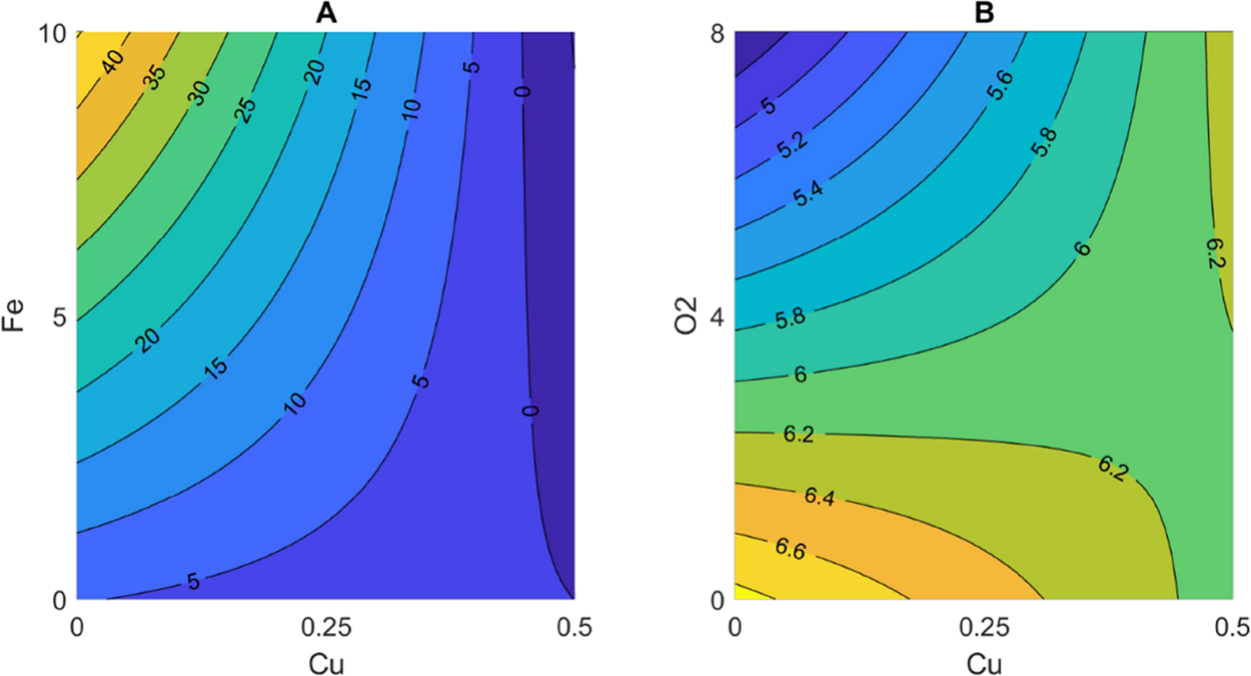
Results from photo-degradation trials carried out in model wine solution added with catechin showing the contour plots for the interaction between oxygen and copper for (A) free methanethiol and (B) cabbage sensory score.

The RSM approach for MW added with catechin enabled the development of regression models that explained free MeSH concentration (*R*^2^ = 76.3%) and the cabbage sensory score (*R*^2^ = 73.0%) (Figure 6A). The reason why only two regression models could explain the influence of variables on the photo-degradation of RF and Met in the presence of catechin could not be readily rationalized. Perhaps the different reactivity of the flavan ring system in flavonoids meant that significant models were unable to be generated to predict the impact of these light-induced reactions, unlike hydroxycinnamates such as caffeic acid. Whatever the reason, the limited formation of VSCs in the presence of catechin appeared clear. For free MeSH, negative regression coefficients were found for copper and the copper and iron interaction (Figure 6B). The latter is shown in Figure 7A: increasing iron can favor the formation of free MeSH, whereas copper could limit it when oxygen is present at an average level (3 mg/L) (Figure 7A). This result suggests that the presence of transition metal combined with the presence of oxygen are of particular importance for the formation of VSCs. In the case of copper, both investigated phenols evidenced a negative contribution, while different behavior was observed for the copper and iron interaction (Figures 4B vs 6B). As already mentioned, the chemical nature of phenolics could be of importance for counteracting the appearance of LST, also in relation to the presence transition metals and oxygen. Cabbage sensory score showed a considerable negative regression coefficient with oxygen and a positive coefficient with the interaction of copper and oxygen (Figure 6C), supporting the poor efficacy of copper in limiting the perception of LST. In fact, a greater increase of the cabbage sensory score can be observed for increasing concentrations of copper, even in the case where oxygen concentration increases (Figure 7B).

In conclusion, transition metals such as iron and copper are well-known catalysts of oxidation reactions and can favor the formation of VSCs. Oxygen can also take part in such reactions, affecting the concentration of VSCs that represent the marker volatiles of the wine fault known as LST. To the best of our knowledge, this is first time that the combined effect of oxygen, iron, and copper on the formation of LST has been investigated. We hypothesized that these variables could influence LST formation in a wine-like solution containing RF and Met. The major purpose was to have an overall picture of the complex photo-degradative mechanisms. In particular, with our study, the influence of iron has been shown for the first time, maybe due to its involvement in Strecker degradation, as it can favor the formation of VSCs besides being involved into the photo-Fenton reaction generating glyoxylic acid. Copper on the other hand is commonly used for the depletion of “reduced” aroma defects, but had only a limited impact in the present case; indeed, it may lead to an increase of VSCs under anoxic condition. Ultimately, different combinations of oxygen, iron, and copper were found to play a very important role in the development of LST since different concentrations of VSCs and cabbage sensory scores were revealed depending on the experimental conditions adopted. This suggests that the presence of oxygen at bottling and the levels of iron and copper should be taken into account along with the concentrations of RF and Met. In particular with regard to Met, its chemical degradation did not lead to the formation of the off-flavor as other compounds could also be originated (e.g., methionine sulfoxide).^35,36^ Nonetheless, in most of the experiments, the degradation of Met was related to the formation of DMDS and DMTS—when the decrease of Met was greater than 0.6 mg/L, more than 10 μg/L DMDS and DMTS were found. On the contrary, these VSCs were lower than 10 μg/L when degraded Met was less than 0.6 mg/L.

The addition of phenolics minimized the decrease of Met, probably due to the ability of phenols to compete with Met, both in the Type I pathway as electron donors to the triplet state RF and in the Type II pathway where they compete with Met in the reaction with singlet oxygen. In terms of sensory effects, however, the modeling showed that phenols affected LST depending on their chemical nature. Under the experimental conditions, lower concentrations of VSCs were in most of the cases found when caffeic acid was added in comparison to catechin, thus potentially limiting the appearance of LST.

Overall, besides the presence of RF and Met, the susceptibility of a wine to develop LST appeared to be related to the presence of transition metals as well as to the different phenols that would ordinarily be present in wine. It may be that wines with a higher content of phenolic acids or phenols that are less easily oxidizable than flavan-3-ols could be less susceptible to the appearance of LST, but this aspect requires further verification. VSCs determined in this study have been reported to be the main compounds responsible for the appearance of LST.^6,25,29^ Nonetheless, we cannot exclude that the other compounds generated from side reaction mechanisms (e.g., photo-Fenton reactions) and/or the interactions among them could have an impact on LST intensity perceived. This hypothesis requires further investigation.

## Supporting Information Available

Concentrations of methionine, methionine sulfoxide, and acetaldehyde determined for model wine solution and model wine solution containing caffeic acid or catechin stored in the dark or exposed to light; odor activity values for methanethiol, dimethyl disulfide, and dimethyl trisulfide determined for model wine solution and model wine solution containing caffeic acid or catechin exposed to light (PDF)

## Abbreviations

*d*_6_-DMS: *d*_6_-dimethyl sulfide
DMDS: dimethyl disulfide
DMTS: dimethyl trisulfide
MeSH: methanethiol
Met: methionine
Met sulfone: methionine sulfone
Met sulfoxide: methionine sulfoxide
RF: riboflavin
