# Supplementary material for: Response Surface Methodology Approach to Evaluate the Effect of Transition Metals and Oxygen on Photo-Degradation of Methionine in a Model Wine System Containing Riboflavin
Source: J Agric Food Chem. 2022 Dec 13;70(51):16347–57. doi: 10.1021/acs.jafc.2c05275 (PMC9937536; doi:10.1021/acs.jafc.2c05275)
Supplement: Supplementary file 1 — jf2c05275_si_001.pdf [file jf2c05275_si_001.pdf]

Supplementary information for

Response Surface Methodology Approach to Evaluate the Effect of Transition Metals and Oxygen on Photo-Degradation of Methionine in a Model Wine System Containing Riboflavin

Daniela Fracassetti,[*,†] Davide Ballabio,[‡] Melissa Mastro,[†] Antonio Tirelli[†] and David W. Jeffery[§]

[†]Department of Food, Environmental and Nutritional Sciences (DeFENS), Università degli Studi di Milano, Via G. Celoria 2, 20133 Milan, Italy

[‡]Department of Earth and Environmental Sciences, University of Milano-Bicocca, Piazza della Scienza 1, 20126 Milan, Italy

[§]Department of Wine Science and Waite Research Institute, The University of Adelaide, PMB 1, Glen Osmond, South Australia 5064, Australia

*Email: daniela.fracassetti@unimi.it. Tel.: +390250316674.

Table of contents

	Page
**Table S1:** Concentrations of methionine, methionine sulfoxide, and acetaldehyde detected in the model wine samples stored in the dark or exposed to light. The average content of riboflavin in the samples stored in the dark was 203 ± 7 µg/L; riboflavin was not detected in the samples exposed to light.	3
**Table S2**: Odor activity values (OAVs) for methanethiol determined for model wine solution (MW) and model wine solution containing caffeic acid or catechin that were exposed to light.	4
**Table S3:** Odor activity values (OAVs) for dimethyl disulfide determined for model wine solution (MW) and model wine solution containing caffeic acid or catechin that were exposed to light.	5
**Table S4:** Odor activity values (OAVs) for dimethyl trisulfide determined for model wine solution (MW) and model wine solution containing caffeic acid or catechin that were exposed to light.	6
**Table S5:** Concentrations of methionine, methionine sulfoxide, and acetaldehyde detected in the model wine containing caffeic acid samples stored in the dark or exposed to light. The average content of riboflavin in the samples stored in the dark was 225 ± 12 µg/L; riboflavin was not detected in the samples exposed to light.	7
**Table S6:** Concentrations of methionine, methionine sulfoxide, and acetaldehyde detected in the model wine containing catechin samples stored in the dark or exposed to light. The average content of riboflavin in the samples stored in the dark was 214 ± 9 µg/L; riboflavin was not detected in the samples exposed to light.	8

Table S1: Concentrations of methionine, methionine sulfoxide, and acetaldehyde detected in the model wine samples stored in the dark or exposed to light. The average content of riboflavin in the samples stored in the dark was 203 ± 7 μg/L; riboflavin was not detected in the samples exposed to light.

Run	Copper (mg/L)	Iron (mg/L)	Oxygen (mg/L)	Methionine (mg/L)		Methionine sulfoxide (mg/L)		Acetaldehyde (mg/L)	
				Dark	Light	Dark	Light	Dark	Light
1	0	0	3	2.99	2.05	nd	nd	nd	nd
2	0.5	0	3	2.88	2.20	nd	nd	nd	nd
3	0	10	3	2.87	2.25	0.15	0.12	nd	nd
4	0.5	10	3	2.84	2.02	nd	0.10	nd	nd
5	0	5	0	3.08	2.07	nd	nd	nd	nd
6	0.5	5	0	3.16	1.85	0.16	0.10	nd	0.41
7	0	5	8	2.87	1.87	nd	nd	nd	7.21
8	0.5	5	8	2.91	2.42	nd	nd	nd	1.48
9	0.25	0	0	4.92	3.62	nd	nd	nd	nd
10	0.25	10	0	2.96	2.02	0.10	0.15	nd	0.41
11	0.25	0	8	2.92	2.19	nd	nd	nd	nd
12	0.25	10	8	2.59	2.23	nd	0.13	< 0.1	1.48
13	0.25	5	3	2.73	1.80	nd	nd	nd	1.13
14	0.25	5	3	2.96	1.90	0.13	0.10	nd	1.13
15	0.25	5	3	2.84	1.91	0.15	0.10	nd	0.77

Table S2: Odor activity values (OAVs) for methanethiol determined for model wine solution (MW) and model wine solution containing caffeic acid or catechin that were exposed to light. OAVs were calculated considering the perception threshold of 0.3 μg/L.[1]

Run	Copper (mg/L)	Iron (mg/L)	Oxygen (mg/L)	MW	MW + caffeic acid	MW + catechin
1	0	0	3	0.0	0.2	0.0
2	0.5	0	3	0.6	0.2	0.2
3	0	10	3	112	4.1	13
4	0.5	10	3	57	1.5	0.2
5	0	5	0	179	6.9	5.0
6	0.5	5	0	57	1.5	0.0
7	0	5	8	63	0.6	6.1
8	0.5	5	8	3.5	0.4	0.0
9	0.25	0	0	3.0	0.2	0.2
10	0.25	10	0	6.5	4.5	1.3
11	0.25	0	8	0.4	0.6	0.0
12	0.25	10	8	11	1.1	0.6
13	0.25	5	3	7.8	2.2	0.2
14	0.25	5	3	3.5	2.6	0.4
15	0.25	5	3	5.6	3.0	0.0

Table S3: Odor activity values (OAVs) for dimethyl disulfide determined for model wine solution (MW) and model wine solution containing caffeic acid or catechin that were exposed to light. OAVs were calculated considering the perception threshold of 20-45 µg/L.[2]

Run	Copper (mg/L)	Iron (mg/L)	Oxygen (mg/L)	MW	MW + caffeic acid	MW + catechin
1	0	0	3	0.24-0.53	0.0	0.0
2	0.5	0	3	2.9-62	0.05-0.10	0.07-0.15
3	0	10	3	0.67-1.5	0.0	0.01-0.02
4	0.5	10	3	1.1-2.5	0.0-0.02	0.01-0.02
5	0	5	0	1.2-2.7	0.05-0.10	0.09-0.20
6	0.5	5	0	2.3-5.2	0.0-0.01	0.0
7	0	5	8	0.10-0.15	0.0	0.0-0.01
8	0.5	5	8	0.10-0.18	0.0	0.0-0.01
9	0.25	0	0	2.7-6.1	0.02-0.18	0.18-0.40
10	0.25	10	0	2.1-4.8	0.0	0.03-0.07
11	0.25	0	8	0.34-0.76	0.0	0.01-0.02
12	0.25	10	8	0.10-0.22	0.0	0.02-0.05
13	0.25	5	3	2.1-4.7	0.0	0.04-0.10
14	0.25	5	3	2.5-5.6	0.05-0.10	0.08-0.18
15	0.25	5	3	2.8-6.3	0.06-0.12	0.12-0.26

Table S4: Odor activity values (OAVs) for dimethyl trisulfide determined for model wine solution (MW) and model wine solution containing caffeic acid or catechin that were exposed to light. OAVs were calculated considering the perception threshold of 0.1 μg/L.[2]

Run	Copper (mg/L)	Iron (mg/L)	Oxygen (mg/L)	MW	MW + caffeic acid	MW + catechin
1	0	0	3	79	0.0	0.0
2	0.5	0	3	177	26	56
3	0	10	3	10.3	0.0	0.0
4	0.5	10	3	48	0.18	0.0
5	0	5	0	56	0.0	3.2
6	0.5	5	0	0.0	0.77	0.1
7	0	5	8	10	0.0	0.0
8	0.5	5	8	6.8	0.0	0.32
9	0.25	0	0	92	13	97
10	0.25	10	0	80	0.18	1.7
11	0.25	0	8	190	1.8	7.8
12	0.25	10	8	2.3	0.0	0.23
13	0.25	5	3	118	0.14	17
14	0.25	5	3	102	0.14	34
15	0.25	5	3	137	0.45	37

Table S5: Concentrations of methionine, methionine sulfoxide, and acetaldehyde detected in the model wine samples containing caffeic acid stored in the dark or exposed to light. The average content of riboflavin in the samples stored in the dark was 225 ± 12 μg/L; riboflavin was not detected in the samples exposed to light.

Run	Copper (mg/L)	Iron (mg/L)	Oxygen (mg/L)	Methionine (mg/L)		Methionine sulfoxide (mg/L)		Acetaldehyde (mg/L)	
				Dark	Light	Dark	Light	Dark	Light
1	0	0	3	3.54	3.23	< 0.1	< 0.1	8.64	9.00
2	0.5	0	3	3.46	3.48	< 0.1	nd	7.57	7.92
3	0	10	3	3.13	3.11	0.14	0.04	15.80	16.87
4	0.5	10	3	3.04	2.92	0.28	0.06	17.58	15.80
5	0	5	0	3.06	2.82	0.68	0.12	14.72	16.87
6	0.5	5	0	3.25	2.64	0.55	0.54	15.08	13.65
7	0	5	8	3.49	3.47	nd	nd	13.29	20.80
8	0.5	5	8	3.27	3.16	nd	nd	14.36	15.08
9	0.25	0	0	3.09	2.91	nd	nd	7.57	8.28
10	0.25	10	0	3.05	2.91	nd	nd	17.94	18.30
11	0.25	0	8	2.85	2.74	< 0.1	nd	7.21	8.28
12	0.25	10	8	2.61	2.50	0.18	nd	16.87	19.02
13	0.25	5	3	2.65	2.55	0.32	0.22	12.57	14.72
14	0.25	5	3	2.46	2.33	0.43	0.26	12.93	15.44
15	0.25	5	3	2.29	2.18	0.50	0.39	12.01	14.01

Table S6: Concentrations of methionine, methionine sulfoxide, and acetaldehyde detected in the model wine samples containing catechin stored in the dark or exposed to light. The average content of riboflavin in the samples stored in the dark was 214 ± 9 μg/L; riboflavin was not detected in the samples exposed to light.

Run	Copper (mg/L)	Iron (mg/L)	Oxygen (mg/L)	Methionine (mg/L)		Methionine sulfoxide (mg/L)		Acetaldehyde (mg/L)	
				Dark	Light	Dark	Light	Dark	Light
1	0	0	3	3.57	3.09	nd	nd	8.28	8.64
2	0.5	0	3	3.50	3.12	nd	nd	8.28	9.35
3	0	10	3	3.43	2.99	nd	nd	12.57	12.93
4	0.5	10	3	3.41	2.89	nd	nd	12.22	13.65
5	0	5	0	3.41	3.08	nd	nd	9.00	12.93
6	0.5	5	0	3.42	2.95	nd	nd	11.14	10.79
7	0	5	8	3.25	2.77	nd	nd	10.07	14.01
8	0.5	5	8	3.21	3.18	< 0.1	< 0.1	9.71	11.14
9	0.25	0	0	3.50	3.04	< 0.1	< 0.1	7.57	7.57
10	0.25	10	0	3.50	3.30	< 0.1	0.12	10.79	11.50
11	0.25	0	8	3.47	3.46	< 0.1	0.10	8.64	7.92
12	0.25	10	8	2.49	2.12	0.20	0.19	9.35	11.86
13	0.25	5	3	2.79	2.85	0.24	0.28	10.07	11.14
14	0.25	5	3	3.26	3.18	0.26	0.28	9.71	14.36
15	0.25	5	3	3.03	2.93	0.30	0.30	11.14	10.07

References

[1] Fracassetti, D.; Limbo, S.; Pellegrino, L.; Tirelli, A. Light-induced reactions of methionine and riboflavin in model wine: Effects of hydrolysable tannins and sulphur dioxide. *Food Chem.* **2019**, *298*, 124952.

[2] Fracassetti, D.; Vigentini, I. Occurrence and analysis of sulfur compounds in wine. In *Grapes and wines – advances in production, processing, analysis and valorization*. URL (https://www.intechopen.com/chapters/58638). (Last accessed on 25 July 2022).
